# Exogenous misfolded protein oligomers can cross the intestinal barrier and cause a disease phenotype in *C. elegans*

**DOI:** 10.1038/s41598-021-93527-8

**Published:** 2021-07-13

**Authors:** Michele Perni, Benedetta Mannini, Catherine K. Xu, Janet R. Kumita, Christopher M. Dobson, Fabrizio Chiti, Michele Vendruscolo

**Affiliations:** 1 Centre for Misfolding Diseases, Department of Chemistry, University of Cambridge, Cambridge, CB2 1EW UK; 2 Department of Experimental and Clinical Biomedical Sciences, University of Florence, Florence, Italy

**Keywords:** Protein aggregation, Animal disease models

## Abstract

Misfolded protein oligomers are increasingly recognized as highly cytotoxic agents in a wide range of human disorders associated with protein aggregation*.* In this study, we assessed the possible uptake and resulting toxic effects of model protein oligomers administered to *C. elegans* through the culture medium*.* We used an automated machine-vision, high-throughput screening procedure to monitor the phenotypic changes in the worms, in combination with confocal microscopy to monitor the diffusion of the oligomers, and oxidative stress assays to detect their toxic effects. Our results suggest that the oligomers can diffuse from the intestinal lumen to other tissues, resulting in a disease phenotype. We also observed that pre-incubation of the oligomers with a molecular chaperone (αB-crystallin) or a small molecule inhibitor of protein aggregation (squalamine), reduced the oligomer absorption. These results indicate that exogenous misfolded protein oligomers can be taken up by the worms from their environment and spread across tissues, giving rise to pathological effects in regions distant from their place of absorbance.

## Introduction

Numerous human disorders, including Alzheimer’s and Parkinson’s diseases are associated with the aberrant aggregation of proteins^1–5^. In most cases, these disorders are still incurable, in part because of the incomplete understanding of the mechanism of formation and spreading of protein aggregates. Among the wide variety of misfolded and aggregated protein species, small oligomeric forms, are increasingly recognized to be highly cytotoxic^6–14^. These oligomers are produced during the aggregation process through a variety of complex processes^15,16^, and it has been shown that they undergo a conversion from disordered to more ordered forms and subsequently mature into amyloid fibrils^16^.

In particular, different types of α-synuclein oligomers have been reported under different conditions^17–22^, including two stable forms, denoted type A and type B, being non-toxic and toxic, respectively^23–26^. Type A oligomers have no detectable β-sheet structure, while type B oligomers have a level of such structure intermediate between that of monomers and fibrils^23–26^. It has been shown in particular that the toxic type B oligomers are able to anchor, through their flexible N-termini, to the membrane, which is then penetrated and destabilized through their structured β-sheet core^25^. Similarly, the N-terminal domain of HypF (HypF-N) from *E. coli* has also been shown to form model toxic and non-toxic oligomeric species^27–29^.

We describe here an investigation of the mechanisms of toxicity associated with oligomeric species from α-synuclein and HypF-N, using the nematode worm *C. elegans*. Our choice of *C. elegans* was based on its proven versatility for whole-organism high-throughput genetic^30–34^ and drug^35–37^ screening. The worms are small (1 mm in length), transparent, and easy to manipulate^38^. In addition, their cellular complexity is comparable to that of higher organisms^34,38^ and their short life-span of about 3 weeks facilitates the rapid study of a wide range of biological phenotypes^38^. Indeed, *C. elegans* has been employed as a model organism for studying the phenotypic effects of protein aggregation^39–42^, for the identification of age-related genes and genetic pathways, and for screening protein aggregation inhibitors^40,43,44^.

In this work, we established a protocol to deliver the oligomers to *C. elegans,* investigated how the oligomers were adsorbed and then monitored the resulting effects of the oligomers on the overall fitness of the worms.

## Results

### Delivery of protein oligomers to *C. elegans*

The metastable and heterogeneous nature of misfolded protein oligomers makes it challenging to study their structures and properties and to understand at the molecular level the ways in which they interact with the components of biological systems^2,14,16^. In order to address such questions, we started our investigations by considering model oligomers formed by HypF-N. HypF-N forms spherical oligomers and amyloid-like fibrils in vitro, under conditions that destabilize its native structure^45–48^. Furthermore, when added to the extracellular medium of cultured cells or injected into rat brains, the oligomers induce similar biochemical, electrophysiological, and cell biological responses to oligomers associated with neurodegenerative diseases^6,28,29,49–54^. HypF-N oligomers represent powerful research tools, as HypF-N can be converted into two different types of oligomers, denoted type A and type B, where the former is toxic and the latter is not^27–29^. Both types are sufficiently stable to allow detailed structural and biological studies to be carried out^27–29,51,53–55^. These two oligomeric species have similar sizes and morphologies, and both have a high content of β-sheet structure and the ability to bind the amyloid-specific dye thioflavin T (ThT) to a moderate but significant extent^27,55^.

**Figure 1:**
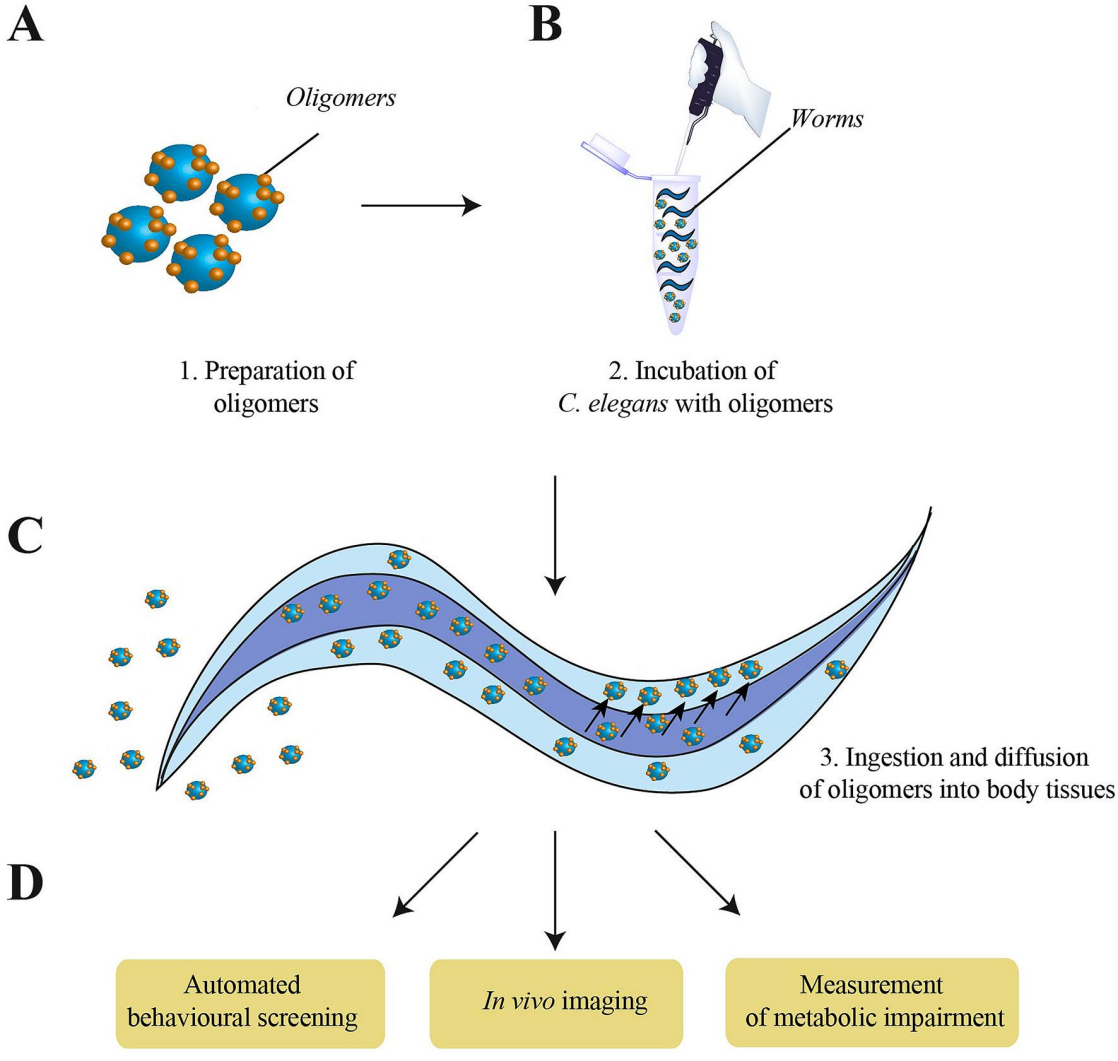
Schematic description of the protocol to probe the effects of exposure of *C. elegans* to misfolded protein oligomers. (**A**) Oligomers were formed as described previously^23,24,27^. (**B**) Worms were incubated at day 0 (D0) with the different oligomeric species for 6–12 h. (**C**) Diffusion of labeled oligomers through the intestinal barrier was monitored through confocal microscopy. (**D**) High-throughput strategies were used to screen for worm fitness after exposure.

We added toxic type A and non-toxic type B HypF-N oligomers, prepared as described previously (see Methods), to the worm growth media at concentrations of 6–48 μM, so that the worms were continuously exposed to the oligomers (Fig. 1). We found the optimal parameters for these experiments to be a density of about 1.2 worms μl^−1^, a duration of incubation of 6–12 h, a volume of media of 750 μl in a 1.5 ml tube to avoid suffocation of the worms and a degree of agitation of 60 rounds per minute (rpm) at 20 °C. We used Eppendorf tubes rather than solid media or multi-well plates in order to minimize the stress to the animals while at the same time increasing their exposure to the oligomers. We also tested a variety of different media compatible with the growth of *C. elegans*, including Hepes, phosphate-buffered saline (PBS), plain water and M9 buffer at physiological pH 7.4, and a range of different ages of the worms for oligomer administration and screened their fitness with a completely automatically procedure^56^.

To optimize the exposure of the worms to the oligomers, we found advantageous to perform the incubation in solution, rather than on agar plates where the oligomers were more easily degraded or avoided by the animals. We screened the behavior of the worms by means of a recently developed automated high throughput platform^56^, and examined about 600 worms for each condition used in this study.

### Type A and type B HypF-N oligomers exhibit different toxicities in *C. elegans*

**Figure 2:**
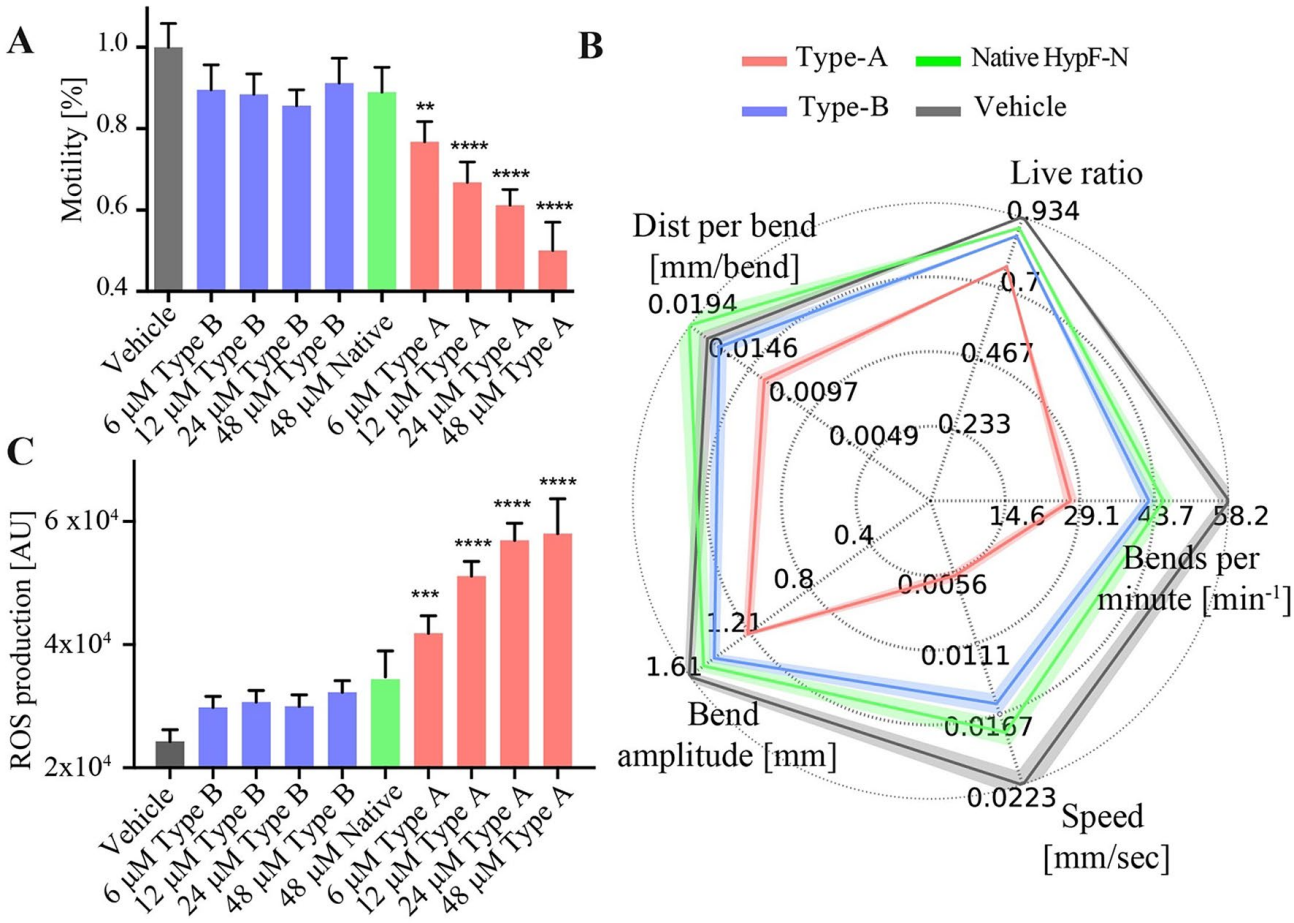
Effects of early exposure to HypF-N oligomers on worm fitness. (**A)** Type A (red) and type B (blue) oligomers of HypF-N^27^, at 6, 12, 24, 48 μM and native HypF-N at 48 μM (green) were administered to *C. elegans* at day 0 (D0). 600 animals were analyzed in each experiment, and a representative of three replicas, which showed similar results, is shown. Statistical tests (Student’s t test) were carried out using Graph-pad prism. All tests have been two tailed unless stated otherwise. Error bars indicate the standard error of the mean (SEM). The double (**), triple (***) and quadruple (****) asterisks indicate P ≤ 0.01, 0.001 and 0.0001, respectively, relative to unexposed worms. (**B**) High resolution phenotypical fingerprinting carried out using WF-NTP. Multiple fitness readouts (speed, bends per minute, bend magnitude, distance per bend and live ratio) are reported. Up to 1200 animals per condition were analyzed. Thick colored lines indicate the mean and SEM values. (**C**) ROS production in worms exposed at D0 with type A (red) and type B (blue) oligomers of HypF-N^27^, at 6, 12, 24, 48 μM and native HypF-N at 48 μM (green) Bars indicate the means and SEM values.

Type A HypF-N oligomers were found to cause significant changes to the motility of the worms at all concentrations tested, and also to show a marked dose dependence of the effects (Fig. 2A). By contrast, type B oligomers and native proteins were found to have no detectable effects at any of the concentrations studied in this work (6–48 μM and 48 μM respectively) (Fig. 2A). Moreover, we observed that type A oligomers affected significantly other characteristics of the behavior of the worms, including swimming speed and rate of paralysis, while the type B and monomeric native HypF-N showed only marginal effects on any of these parameters. The data are summarized (Fig. 2B) by multi-parametric fingerprinting maps, which identify multiple metrics in parallel and define a total fitness score.

We found that the effects of the oligomers were most evident following their early administration at day 0 (D0) of adulthood with 750 μl of M9 buffer at pH 7.4 (Fig. 2A, the motility is normalized to the value of the vehicle). Administration at day 4 (D4) of adulthood showed less pronounced effects, which were only evident for type A HypF-N oligomers and only at the highest concentrations tested (> 24 μM) (Figure S1).

We also measured production of reactive oxidative species (ROS) using a luminometer (Methods). After incubation times of up to 6 h, a significant production of ROS and a clear dose dependence was observed with type A oligomers, whereas the effects of type B oligomers and native HypF-N were found to be negligible and non-significant (Fig. 2C).

### Toxic HypF-N oligomers are adsorbed from the guts in *C. elegans*

**Figure 3:**
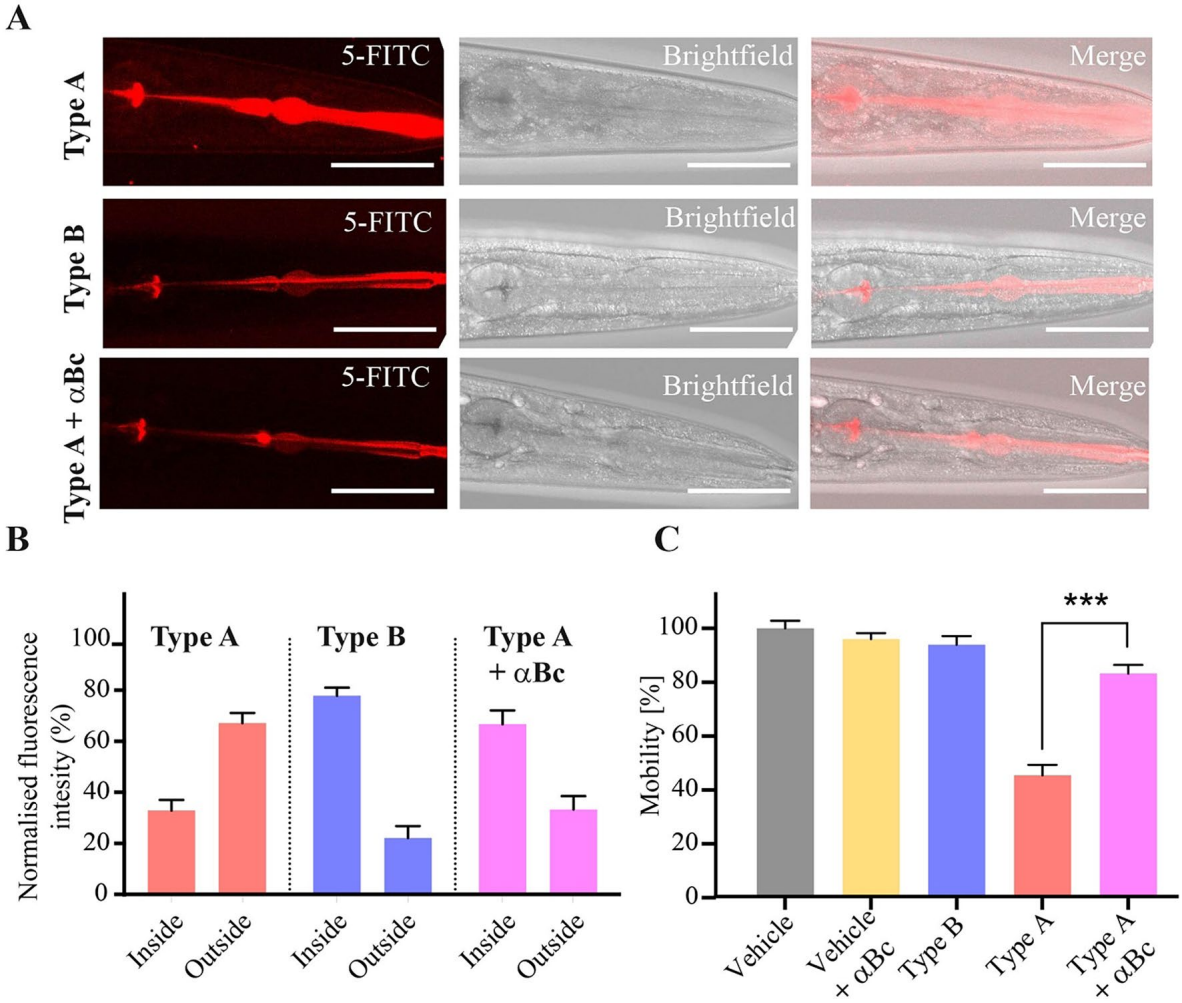
Oligomer uptake from the intestinal lumen to the internal tissues of the worms. (**A**) Representative images of oligomer localisation obtained by confocal microscopy. Horizontal bars in the images indicate 100 μm. (**B**) Quantification of uptake of the oligomers from the intestinal lumen (inside) to the internal tissues (outside) of the worms. Worms were exposed to 12 µM of type A, or type B oligomers or with type of A oligomers with αB-crystallin (αBc) at a 5:1 HypF-N:αB-crystallin molar ratio (Type A + αBc) at D0. HypF-N was labeled with 5-FITC or unlabeled. All oligomers were incubated with 300 wild-type worms over a period of 12 h, after which confocal microscopy images were acquired. Bars indicate mean and SEM values. (**C**) Analysis of the motility of worms exposed at day 0 (D0) to 48 µM type B (blue), type A (red), type A oligomers with 9.6 μM αB-crystallin (pink) or left unexposed (“vehicle”, grey) and treated with αB-crystallin only (“vehicle + αBc”, yellow) and analyzed after 6 h of incubation. Bars indicate mean and SEM values. One representative of three experiments, all of which showed similar results, is shown. Statistical tests (Student’s t test) were carried out using Graph-pad prism. The triple (***) asterisks indicate P ≤ 0.001, relative to worms exposed to type A oligomers.

**Figure 4:**
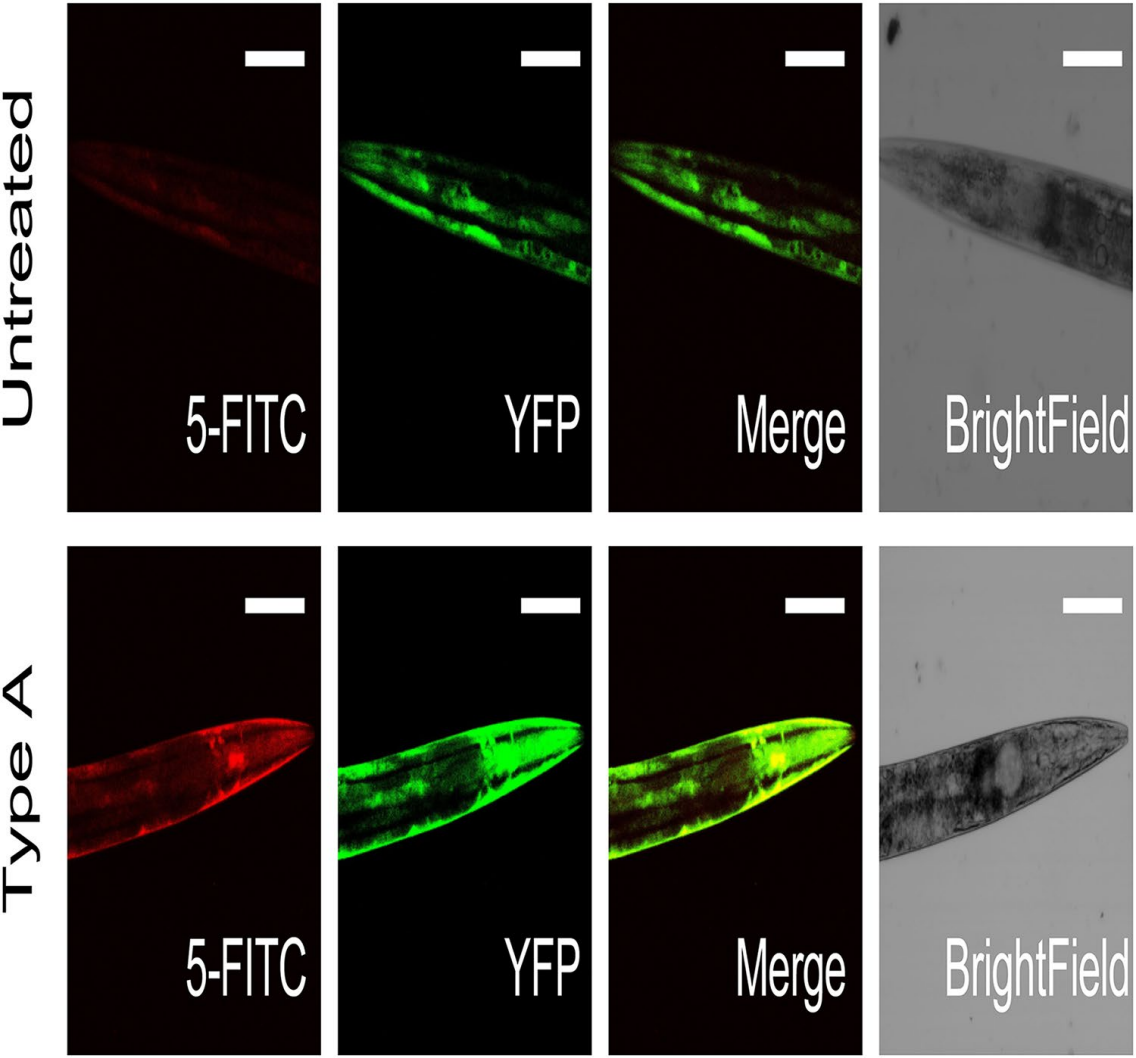
Confocal microscopy analysis of HypF-N type A oligomer co-localisation with muscle cells. Analysis by confocal microscopy of worms expressing YFP in the body wall muscle cells^46^ left untreated (top panels) or exposed to 12 µM of type A oligomers (bottom panels). 300 wild-type worms were exposed over a period of 16 h, after which confocal microscopy images were acquired; horizontal bars indicate 80 µm.

We next sought to elucidate the mechanism by which toxic type A HypF-N oligomers are able to exert their detrimental effects on *C. elegans*. For this purpose, we screened HypF-N oligomers fluorescently labeled with the dye fluorescein-5-isothiocyanate (5-FITC), and acquired confocal microscopy images to evaluate their localization within the worm bodies (Figs. 3A, 4). Measurement of the intensity of the fluorescence signals showed that both type A and type B oligomers were rapidly ingested by the worms, and in both cases the oligomers were located in the intestinal lumen after 2 h of incubation. After longer incubation times (from 6 to 12 h), however, substantial differences were observed between these two types of oligomers. These results suggest that type A oligomers are able to pass through the intestinal lumen and to diffuse into the internal tissues of the worms. By contrast, type B oligomers appear to remain in the intestinal lumen even after 12 h of incubation (Fig. 3A) where they are likely to be slowly degraded.

Quantification of the fluorescence intensities arising from the labeled oligomers was then carried out to compare their levels in the intestinal lumen and in nearby tissues (Fig. 3B) In the case of type B oligomers, we found that after 12 h of incubation the majority of the fluorescently tagged oligomers (78%) were localized within the intestinal lumen of the animals. In the case of type A oligomers, we observed that the vast majority of the signal (67%) came from the adjacent tissues with only 33% in the intestinal region (Fig. 3B). The phenotypic analysis indicates that the motility of the worms was reduced only when the animals were exposed to type A oligomers (Fig. 2A,B).

These results are consistent with the possibility that the type A oligomers could penetrate the intestinal cells of the worms, and that this causes widespread toxicity. Type B oligomers, by contrast, appear unable to disrupt to any significant degree the integrity of the cells of the intestine, in which they remain confined and where eventually they are degraded, an effect likely to be a result of continuous exposure to the highly acidic pH of the intestinal lumen^57^. The different ability of the two types of oligomers to interact with cell membranes has previously been shown to be responsible for differential toxic effects in cultured neuroblastoma cells where only type A oligomers were found to disrupt cellular membranes^27,29^. The set of confocal microscopy images reported here is consistent with the hypothesis that an important mechanism of action of oligomeric aggregates associated with perturbed motility involves the impairment of the integrity of cellular membranes^25^.

We then carried out experiments to investigate if the HypF-N type A oligomers localize in the body wall muscle cells of the worms. In order to investigate this point, we used worms that express the yellow fluorescent protein (YFP) in the body wall muscle cells^46^ and administered to them type A oligomers tagged with 5-FITC (Fig. 4). Despite the relatively low intensity of the 5-FITC signal, there was a clear colocalization between the labelled oligomers and the YFP, indicating that the oligomers reach the muscle cells from the gut lumen. Interestingly, we could also observe an increased fluorescent signal arising from the muscle tissue when the worms were administered fluorescent type A oligomers, suggesting that the muscle cells also change their levels of protein expression (Fig. 4). These results suggest accumulation of the toxic species in the body wall muscle cells, although the oligomers seem to diffuse also to other tissues within the body of the worms (Figure S2).

### αB-crystallin reduces the toxicity of HypF-N oligomers

In order to explore further the link between the location of the oligomers within the worms and their toxicity, and to assess the degree by which such toxicity arises from the ability of these species to cross the intestinal cell walls, we also pre-incubated type A HypF-N oligomers with αB-crystallin at a molar ratio of 5:1 for 1 h before administering them to the worms and incubating for 12 h (Fig. 3A–C). αB-crystallin is a molecular chaperone that has been shown to be able to reduce the toxicity of HypF-N type A oligomers in cell cultures by promoting their assembly into species that cannot readily pass through the cell wall^52,63^. Following exposure of the worms with type A oligomers pre-incubated with this molecular chaperone, the motility of the worms was substantially higher than that of the worms exposed to type A oligomers in the absence of αB-crystallin. Indeed, it was found to be similar to that of the worms exposed to the medium alone or to type B oligomers (Fig. 3C). In addition, the fluorescence data showed that the type A oligomers treated with αB-crystallin remained overwhelmingly within the intestinal lumen (Fig. 3A), providing further evidence of the link between the toxicity of the oligomers and their ability to diffuse through the intestinal cell walls.

### α-Synuclein oligomers reduce worm motility and increase ROS production

**Figure 5:**
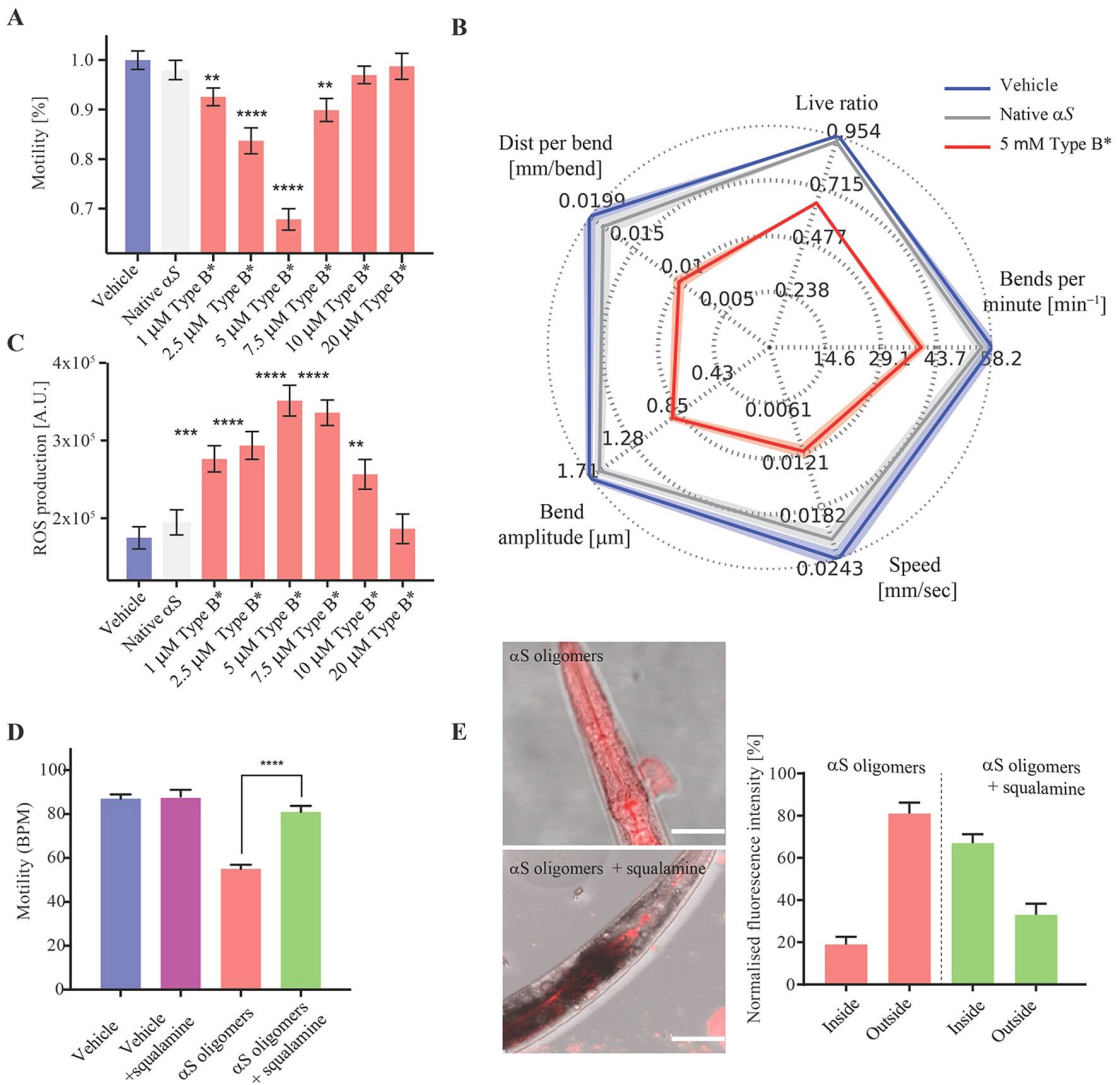
Effects of the exposure to type B α-synuclein oligomers on the worm fitness. (**A**) Worms were exposed at day 0 (D0) of adulthood to type B α-synuclein (αS) oligomers^23,24^ at 1, 2.5, 5, 7.5, 10 and 20 μM (monomer equivalents), the motility is normalized to the value of the vehicle. 600 worms were analyzed after 6 h exposure per condition. Bars indicate mean and SEM values. One experiment representative of three, which showed similar results, is shown. Statistical tests (Student’s t test) were carried out using Graph-pad prism. The double (**), triple (***) and quadruple (****) asterisks indicate P ≤ 0.01, 0.001 and 0.0001, respectively, relative to unexposed worms. (**B**) High resolution phenotypical fingerprinting carried out using the wide field of view nematode tracking platform (WF-NTP, colors as in (**A**). Multiple fitness readouts (speed, bends per minute, bend magnitude, distance per bend and live ratio) are reported. Up to 1200 animals per condition were analyzed. Thick coloured lines indicate mean and SEM values. (**C**) ROS production in worms exposed at day 0 (D0) to α-synuclein oligomers at 1, 2.5, 5, 7.5, 10 and 20 μM (monomer equivalents). Bars indicate the means and SEM values. (**D**) Quantification of the worm behavior after exposure at D0 to 2.5 µM α-synuclein oligomers without or with 10 µM squalamine. Bars indicate mean and SEM values. Approximately 600 worms were analysed after incubation for 6 h. (**E**) Confocal microscopy images of worms exposed at D0 with 2.5 μM α-synuclein oligomers labeled with 5-FITC (right) or without (left) 12.5 µM squalamine at a α-synuclein:squalamine molar ratio of 1:5. The images were taken after 12 h of incubation. Horizontal bars indicate 80 μm.

In order to study directly α-synuclein oligomers, we exposed the worms to toxic type B oligomers. We previously reported that the exposure to such oligomers was highly toxic to human SH-SY5Y neuroblastoma cells and primary neurons, as observed from an increase in ROS, Ca^2+^ uptake and calcein release through disruption of cellular membranes and a decrease in mitochondrial activity^23,25,58,59^. In those studies, we observed a decrease in toxicity, not just at lower α-synuclein concentrations, but also at higher concentrations, an effect that can be attributed to the further aggregation (agglutination) of the oligomers into large assemblies^60^, a phenomenon typical of hydrophobic colloids, such as our oligomers, in water solutions. In the present work, α-synuclein oligomers were administered to the worms at the D0 stage and were observed to have effects similar to those of the type A HypF-N oligomers, with the maximum effect on motility (Fig. 5A,B) and ROS (Fig. 5C) production observed at a concentration of 5 μM (monomer equivalents). The dose dependence was similar to that observed with the neuroblastoma cells, except that the concentration of maximum toxicity in the worms was some ten times higher than that in the cells, suggesting that the effective concentration that was established within the worm bodies was lower than in the case of the cell cultures. These results indicate that the toxic effects observed in *C. elegans* for the HypF-N oligomers are also found for the disease-related oligomeric species of α-synuclein, that are associated with the pathogenesis of PD.

### Treatment with squalamine reduces the toxicity of type B α-synuclein oligomers

In a series of additional experiments, the toxic type B oligomers of α-synuclein were preincubated with the aminosterol squalamine (Fig. 5D,E), which was shown to have a substantial protective effect against the toxicity of these oligomers in neuronal cells and when formed following overexpression in *C. elegans*^56^. A similar protective effect against the toxicity of the same oligomers in neuronal cells was also found for the structurally similar molecule trodusquemine, which has a spermine moiety rather than a spermidine group^61^. The results showed that this procedure reduced the deleterious effects of the addition of pre-formed α-synuclein oligomers (Fig. 5D), and confocal microscopy confirmed that the administration of α-synuclein oligomers incubated with squalamine greatly reduced their capacity to permeate the gut walls (Fig. 5E).

## Discussion

We have described the toxic effects of protein oligomers administered exogenously in the model organism *C. elegans.* We first studied oligomers of the model protein HypF-N, from which stable oligomers that are both toxic (type A) and non-toxic (type B) to neuronal cells in culture can be generated^27–29,51,53–55^ by adding them to the worm culture medium. We then evaluated the effects on the worms exposed to these species by means of an automated tracking platform^56^, which enabled high-throughput and highly sensitive measurements of the effects on the fitness of the animals to be made^56,62–64^.

We observed a high level of toxicity in the worms incubated with type A HypF-N oligomers, with negligible effects observed following incubation with the type B or the native HypF-N protein. We also measured a higher oxidative stress in the worms exposed to type A oligomers relative to those exposed to type B oligomers or native proteins, where the observed effects were very small. We further observed that only type A oligomers were capable of passing through the intestinal membrane and diffusing into nearby tissues. This result shows the strong link between the properties of the intestinal cells of the worm and their health and lifespan^65,66^*,* pointing at the importance of this entry route for oligomeric species in this system. Unlike type A oligomers, type B oligomers were found to be unable to penetrate the membranes of the cells of the intestinal wall and to cause significant detrimental effects. This difference in behavior of the two types of oligomers observed in this animal model system correlates with that observed previously in cell cultures^27–29,51,53–55^ supporting the conclusion that the interaction with cell membranes plays a central role in determining the toxicity of the oligomers. Indeed, it was previously found that toxicity of misfolded protein oligomers to cell cultures correlates with both exposure of hydrophobic groups and small size of the oligomers, which favors their rapid diffusion through the lipid bilayer of cell membranes^27–29,51,53–55,67^, a finding that is confirmed in the present report showing an ability of the oligomers to diffuse through such the membranes. This finding was reinforced further by incubating the HypF-N oligomers with the molecular chaperone αB-crystallin, which has previously been shown to be protective against the toxicity of the type A oligomers in a cellular system^50^. In the presence of αB-crystallin a marked increase in the fitness of the worms was observed, relative to that in the absence of the molecular chaperone.

We then extended these results to a type of α-synuclein oligomers that have been shown previously to be toxic to cultured cells^23–25,58,59^. We observed dose-dependent toxic effects similar to those found in cultured neuroblastoma cells and primary neurons. Furthermore, incubation with small therapeutic molecules, such as the aminosterol squalamine, found previously to be protective in cell models and in transgenic PD strains of *C. elegans* by binding to the cell membrane and displacing oligomeric α-synuclein^56^, suppresses dramatically the oligomeric toxicity in this system. Given that the populations of oligomers produced during aggregation reactions is highly heterogeneous in conformational and physico-chemical properties^68^, the results that we have reported for HypF-N and α-synuclein oligomers provide further insight into the relationship between these properties and cytotoxicity^25,53,69,70^.

In a broader context, it has been suggested that Parkinson’s disease may in certain cases originate in the gastrointestinal tract before progressing to the brain^71–73^. Accumulation of α-synuclein has been observed in the enteric nervous system (ENS), which spans the entire gastrointestinal tract, as well as in the stomach, duodenum and colon of PD patients and healthy individuals^74–76^. In this mechanism of disease, pathological forms of α-synuclein may ascend ENS-innervating vagal fibers to the nodose ganglion and brainstem nuclei^71^. Indeed, it has recently been reported that inoculation of preformed fibrils of α-synuclein in the duodenal intestinal lining of ageing mice can lead to brain pathology^77,78^. It has also been suggested that amyloid aggregates from the gut flora in mice could cross the gut barrier and trigger α-synuclein in the ENS^79,80^. Although the mechanism of formation of α-synuclein aggregates in the human ENS has yet to be fully clarified, our results provide additional insight into the hypothesis that α-synuclein aggregates originating from the gut can spread to other regions of the body.

## Conclusions

We have shown that misfolded protein oligomers have different abilities to gain access from the gut lumen to specific types of tissues in *C. elegans*. This differential uptake can be linked to the ability of certain types of exogenous oligomers of environmental origin to cross the intestinal barrier and to diffuse through the internal tissues of the worms. As these processes can be linked to the ability of the most cytotoxic oligomers to cross cell membranes, these results provide a glimpse on the importance of aggregate spreading in protein misfolding diseases.

## Materials and methods

### Media

Standard conditions were used for the propagation of *C. elegans*^81–83^. Briefly, the animals were synchronized by hypochlorite bleaching, hatched overnight in M9 buffer (3 g l^−1^ KH_2_PO_4_, 6 g l^−1^ Na_2_HPO_4_, 5 g l^−1^ NaCl, 1 µM MgSO_4_), and subsequently cultured at 20 °C on nematode growth medium plates (NGM, 1 mM CaCl_2_, 1 mM MgSO_4_, 5 µg ml^−1^ cholesterol, 250 µM KH_2_PO_4_, pH 6.0, 17 g l^−1^ Agar, 3 g l^−1^ NaCl, 7.5 g l^−1^ casein), seeded with the *E. coli* strain OP50. Saturated cultures of OP50 were grown by inoculating 50 ml of LB medium (10 g l^−1^ tryptone, 10 g l^−1^ NaCl, 5 g l^−1^ yeast extract) with OP50 and incubating the culture for 16 h at 37 °C. NGM plates were seeded with bacteria by adding 350 µl of saturated OP50 to each plate and leaving the plates at 20 °C for 2–3 days. On day 3 after synchronization, the animals were placed on NGM plates containing 5-fluoro-2′deoxy-uridine (FUDR) (75 µM, unless stated otherwise) to inhibit the growth of offspring.

### Strains of* C. elegans*

We used the *C. elegans* Var Bristol (known as N2), which has a generation time of about 3 days and a brood size of about 350 worms^38^. We also used worms that express the yellow fluorescent protein (YFP) in the body wall muscle cells, known as OW450^46^.

### Preparation of HypF-N oligomers

HypF-N oligomers were obtained as described previously^27^. Briefly, type A and type B monomers were incubated at 48 μM for 4 h at 25 °C under two conditions: (1) 50 mM acetate buffer, 12% (v/v) trifluoroethanol (TFE), 2 mM dithiothreitol (DTT), pH 5.5 (condition A) and (2) 20 mM trifluoroacetic acid (TFA), 330 mM NaCl, pH 1.7 (condition B). The oligomers were centrifuged at 16,100 g for 10 min, dried under N_2_ and resuspended in M9 buffer at the desired concentration. Type A oligomers^50^ were resuspended also in M9 buffer at a concentration of 48 µM (monomer equivalents) with αB-crystallin (Abcam PLC, Cambridge, UK) at the HypF-N-to-chaperone molar ratio of 5:1 for 1 h at 37 °C while shaking. As a control, samples containing the same concentration of αB-crystallin was incubated under the same conditions, in the absence of the oligomers, and then administered to the worms.

### Purification of α-synuclein

α-Synuclein protein expression, purification and oligomer formation were carried out as described previously^24^. Briefly, α-synuclein was purified from *Escherichia coli* BL21 cells overexpressing α-synuclein, which were lysed by sonication. Heat-sensitive proteins were precipitated out of the lysate supernatant by boiling. Following centrifugation, streptomycin sulphate was added to the supernatant and centrifuged again. α-Synuclein was precipitated out of the supernatant by addition of ammonium sulphate (361 mg ml^−1^) and stirring at 4 °C for 30 min. The precipitated α-synuclein was resuspended 25 mM Tris buffer, pH 7.4, 20 °C and loaded onto a HiLoad 26/10 Q Sepharose high performance column (GE Healthcare Ltd., Little Chalfont, UK), and eluted at ~ 350 mM NaCl, 20 °C with a salt gradient from 0 to 1.5 M NaCl. Selected fractions were subsequently loaded onto a Superdex 75 26/60 (GE Healthcare Ltd.) at 20 °C and eluted in PBS, pH 7.4, 20 °C. Protein concentration was determined by absorbance at 275 nm, using an extinction coefficient of 5600 M^−1^ cm^−1^.

### Preparation of α-synuclein oligomers

Samples enriched in oligomeric α-synuclein species were prepared as previously described^24^. Briefly, monomeric α-synuclein was lyophilized in Milli-Q water and subsequently resuspended in PBS, pH 7.4, 20 °C, to give a final concentration of ca. 800 µM (12 mg ml^−1^). The resulting solution was passed through a 0.22 µm cut-off filter before incubation at 37 °C for 20–24 h under quiescent conditions. Small amounts of fibrillar species formed during this time were removed by ultracentrifugation for 1 h at 90,000 rpm (using a TLA-120.2 Beckman rotor, 288,000 g). The excess monomeric protein and some small oligomers were then removed by multiple filtration steps using 100-kDa cut-off membranes. The final concentration of oligomers was estimated on the basis on the absorbance at 275 nm using a molar extinction coefficient of 5600 M^−1^ cm^−1^.

### Delivery of the oligomers

About 600 worms were incubated in M9 buffer with appropriate concentrations of protein oligomers or native proteins in a final volume of 750 μl in 1.5 ml low binding Eppendorf tubes up to 6–12 h under mild shaking (60 rpm) in horizontal position at room temperature. After incubation, the worms were plated on agar and the parameters defining their fitness were analyzed in an automated manner^56,84^. Motility procedures were carried out after 6 h incubation, and imaging was performed after 24 h after incubation. All experiments were carried out in triplicate. One experiment in each case that is representative of the three independent measurements is shown in the figures. We note that the present protocol differs from a previously proposed one^85^, as in our case we do not feed the worms with *E. coli* engineered to express the oligomerizing protein.

### Image analysis

Image analysis was carried out as previously described^27^. Briefly, we used a custom software written in Python (Python Software Foundation), which is part of the WF-NTP platform^56,84^. This code initially detects and subtracts the background, consisting of non-moving objects such as small particles and shadows from the agar plate. After this operation, the remaining labeled regions are identified as individual worms and the positions of said regions are then stored for each frame. The eccentricity of each tracked worm, a measure of the ratio of the major and minor ellipse axes, can then be used to estimate worm bending as a function of time. Through this method, individual worms were tracked over time, and plots of their movement extracted to give visual information about their mobility levels.

### Automated motility assay on agar plates

All *C. elegans* populations were cultured at 20 °C and developmentally synchronized from a 4 h egg-lay. At 64–72 h post egg-lay (time zero) individuals were transferred to FUDR plates. At specific ages, the animals were washed off the plates with M9 buffer and incubated with specific oligomers. After 12 h of incubation, the worms were spread over an OP-50 un-seeded 9 cm plate, after which their movements were recorded at 20 fps using a novel microscopic setup for 30 s or 1 min^56^. Up to 600 animals in triplicate were counted in each experiment unless stated otherwise. One experiment that is representative of the three measured is shown in each case. Videos were analyzed using a custom-made tracking code^56,84^. All statistical tests (Student’s t test) were carried out using Graph-pad prism. All tests have been two tailed unless stated otherwise.

### Protein labelling

Monomeric HypF-N was buffer exchanged in phosphate buffered saline (PBS) solution at pH 7.4, 4 °C, or oligomeric α-synuclein prepared in PBS, and then labelled with the probe 5-FITC using the AnaTag 5-FITC Microscale Protein Labeling Kit (AnaSpec, San Jose, CA, USA). The protein:5-FITC molar ratio was 2:1, to reduce the possible impact of the probe on the aggregation process. The degree of labelling was evaluated by measuring the absorbance of the protein at 280 nm and of the dye at 494 nm, according to the manufacturer’s protocol. The labelled proteins were then converted into their corresponding oligomers.

### Confocal microscopy imaging

The fluorescence of 5-FITC-tagged HypF-N and α-synuclein was quantified using a Leica SP8 confocal microscopy system (Leica Microsystems, Mannheim, Germany) at a nominal magnification of 10X or 20X; for each picture we acquired 20 stacks of 0.7 μm/each. Measurements of fluorescence intensity were performed using ImageJ software (National Institutes of Health, Bethesda, MD, USA). At least 30 animals were examined per condition, unless stated otherwise. All experiments were carried out in triplicate and the data from one representative experiment are shown in the figure. The Student’s t test was used to calculate p values, and all tests were two-tailed unpaired unless otherwise stated.

### Colocalization studies

The internal localisation of 5-FITC-tagged HypF-N type A oligomers was followed using a Leica SP8 confocal microscopy system (Leica Microsystems, Mannheim, Germany) at a nominal magnification of 10X; for each picture we acquired 20 stacks of 0.7 μm/each using both the 5-FITC and YFP channels. The resulting images were then merged using the ImageJ software (National Institutes of Health, Bethesda, MD, USA). At least 30 animals were examined per condition. All experiments were carried out in triplicate and the data from one representative experiment are shown in the figure.

### ROS production and measurement

A ROS-Glo H_2_O_2_ cell kit assay was used (Promega, Fitchburg, Wisconsin, USA). The ROS-Glo H_2_O_2_ Assay is a bioluminescent assay that measures the level of H_2_O_2_, a reactive oxygen species (ROS), directly in cell culture or tissue or in defined enzyme reactions. A derivatized luciferin substrate is incubated with sample and reacts directly with H_2_O_2_ to generate a luciferin precursor. We adapted the protocol for *C. elegans* studies as follows: 500 AD worms and 500 control worms exposed to different oligomers, respectively, were collected at the D0 of adulthood and incubated for 6 h with a ROS Substrate Solution (Promega, Fitchburg, Wisconsin, USA), with mild shaking to avoid worms sedimentation, after which they were incubated for 20 min with the detection solution and the luminescence was then measured with a luminescence GloMax Explorer System (Promega, Fitchburg, Wisconsin, USA).

## Supplementary Information

Supplementary Information.
