# Supplementary material for: Exogenous misfolded protein oligomers can cross the intestinal barrier and cause a disease phenotype in C. elegans
Source: Sci Rep. 2021 Jul 13;11:14391. doi: 10.1038/s41598-021-93527-8 (PMC8277765; doi:10.1038/s41598-021-93527-8)
Supplement: Supplementary file 1 — Supplementary Information. [file 41598_2021_93527_MOESM1_ESM.pdf]

Supplementary Information

Exogenous misfolded protein oligomers can cross the intestinal barrier and cause a disease phenotype in *C. elegans*

Michele Perni[1+], Benedetta Mannini[1+], Catherine K. Xu[1+], Janet R. Kumita[1], Christopher M Dobson[1], Fabrizio Chiti[2*] and Michele Vendruscolo[1*]

[1]*Centre for Misfolding Diseases, Department of Chemistry, University of Cambridge, Cambridge CB2 1EW, UK*

[2]*Department of Experimental and Clinical Biomedical Sciences, University of Florence, Florence, Italy.*

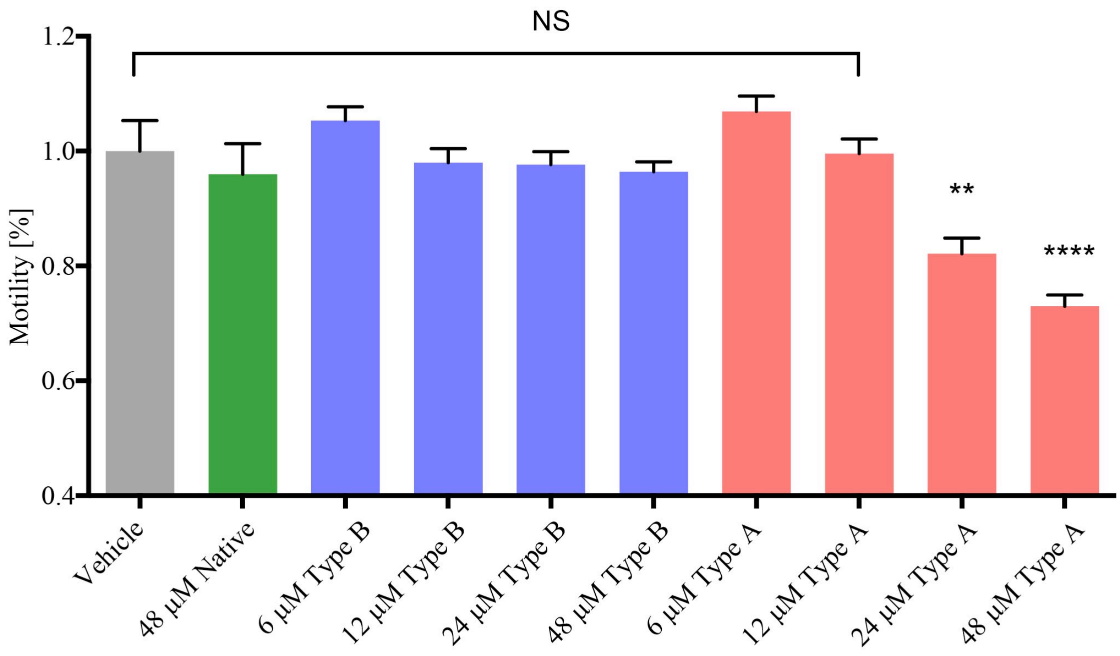

Figure S1. Effects of late exposure to type A and type B oligomers on worm motility. In this experiment, type A and type B HypF-N oligomers[27], at 6, 12, 24, 48 µM and native HypF-N at 48 µM were administered to the animals at D4 of adulthood. Grey, green, blue and red bars represent worms unexposed and exposed with native HypF-N, type B and type A oligomers, respectively. Toxic effects were observed for type A oligomers, but only at a protein concentration >24 µM. 600 animals were analyzed per condition, and one experiment representative of three replicas, all of which showed similar results, is shown. Statistical tests (Student's t-test) were carried out using Graph-pad prism. Error bars indicate the SEM values. The double (**) and quadruple (****) asterisks indicate $P \leq 0.01$ and 0.0001, respectively, relative to unexposed worms.

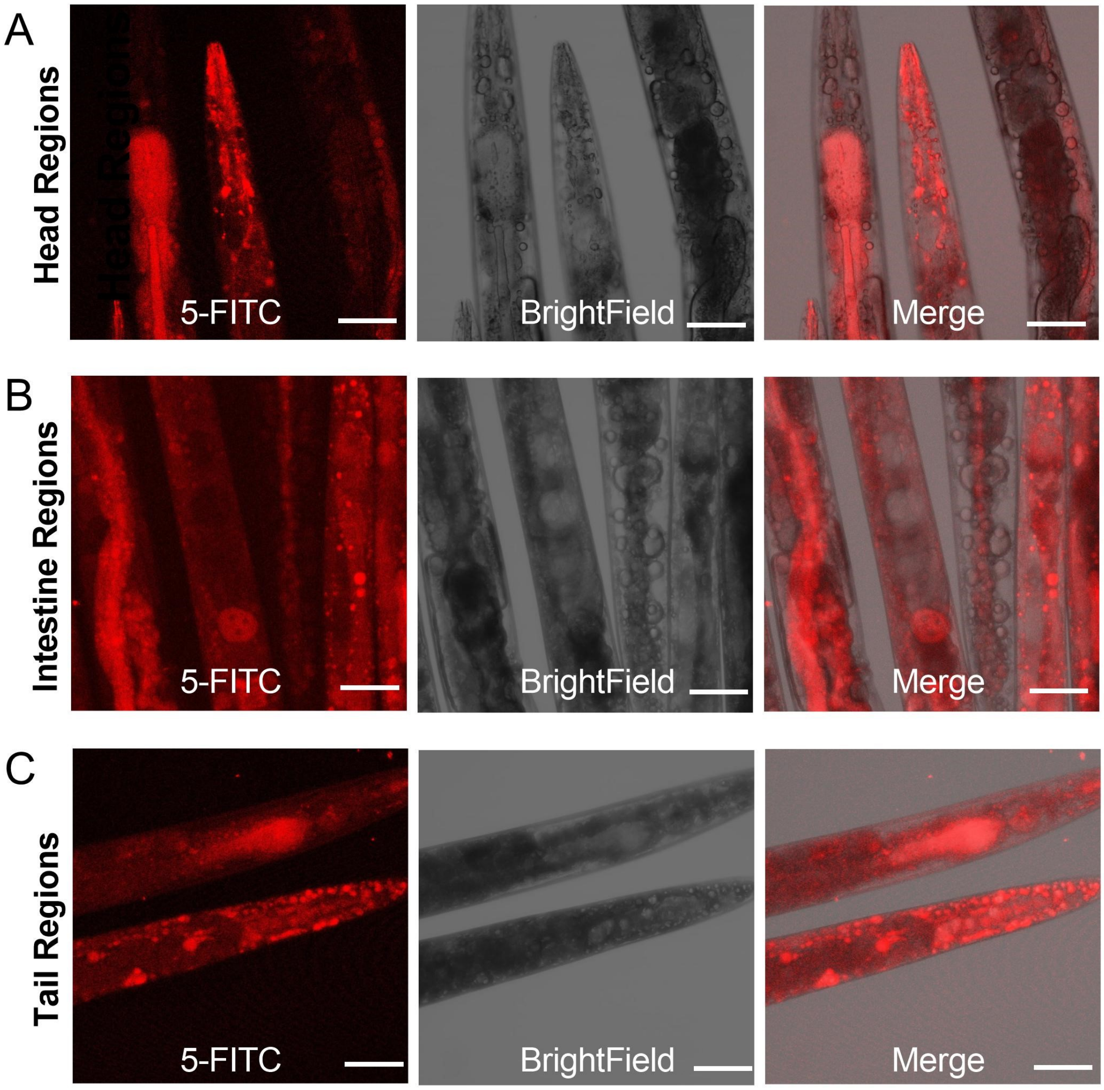

Figure S2. Micrographs of *C. elegans* head, intestines and tail regions showing the HypF-N type A oligomers diffusing to all the worm tissues. Worms were exposed to 12 µM of type A oligomers labeled with 5-FITC. 300 wild-type worms were exposed over a period of 12 h, after which confocal microscopy images of head regions (**A**), intestine regions (**B**) and tail regions (**C**), were acquired. Horizontal bars indicate 80 µm.
